# Plant-Derived Extracellular Vesicles as a Novel Frontier in Cancer Therapeutics

**DOI:** 10.3390/nano14161331

**Published:** 2024-08-08

**Authors:** Lishan Cui, Giordano Perini, Valentina Palmieri, Marco De Spirito, Massimiliano Papi

**Affiliations:** 1 Dipartimento di Neuroscienze, Università Cattolica del Sacro Cuore, Largo Francesco Vito 1, 00168 Rome, Italy; 2 Fondazione Policlinico Universitario A. Gemelli IRCSS, 00168 Rome, Italy; 3 Istituto dei Sistemi Complessi, Consiglio Nazionale delle Ricerche CNR, Via dei Taurini 19, 00185 Rome, Italy

**Keywords:** extracellular vesicles, plant-derived EVs, therapeutic applications, anti-cancer efficacy, drug delivery

## Abstract

Recent advancements in nanomedicine and biotechnology have unveiled the remarkable potential of plant-derived extracellular vesicles (PDEVs) as a novel and promising approach for cancer treatment. These naturally occurring nanoscale particles exhibit exceptional biocompatibility, targeted delivery capabilities, and the capacity to load therapeutic agents, positioning them at the forefront of innovative cancer therapy strategies. PDEVs are distinguished by their unique properties that facilitate tumor targeting and penetration, thereby enhancing the efficacy of drug delivery systems. Their intrinsic biological composition allows for the evasion of the immune response, enabling the efficient transport of loaded therapeutic molecules directly to tumor sites. Moreover, PDEVs possess inherent anti-cancer properties, including the ability to induce cell cycle arrest and promote apoptotic pathways within tumor cells. These vesicles have also demonstrated antimetastatic effects, inhibiting the spread and growth of cancer cells. The multifunctional nature of PDEVs allows for the simultaneous delivery of multiple therapeutic agents, further enhancing their therapeutic potential. Engineering and modification techniques, such as encapsulation, and the loading of therapeutic agents via electroporation, sonication, and incubation, have enabled the customization of PDEVs to improve their targeting efficiency and therapeutic load capacity. This includes surface modifications to increase affinity for specific tumor markers and the encapsulation of various types of therapeutic agents, such as small molecule drugs, nucleic acids, and proteins. Their plant-derived origin offers an abundant and renewable source to produce therapeutic vesicles, reducing costs and facilitating scalability for clinical applications. This review provides an in-depth analysis of the latest research on PDEVs as emerging anti-cancer agents in cancer therapy.

## 1. Introduction

Cancer persists as a prominent and urgent global health concern, ranking among the primary causes of morbidity and mortality worldwide [1,2]. Traditional cancer treatment methods, including chemotherapy and radiotherapy, are commonly employed in clinical settings and have significantly contributed to the extension of human life [3,4]. Even though these approaches do effectively target cancer cells, they lead to substantial side effects including cardio-, neuro-, gastro-, and nephrotoxicity, myelosuppression, mucositis, and alopecia, that further impact the patient’s quality of life [5,6,7]. Given these shortcomings, there is an urgent need for alternative strategies that can selectively target cancer cells while preserving healthy cells to minimize treatment-related toxicities, ultimately improve the overall well-being of cancer patients. 

**Figure 1:**
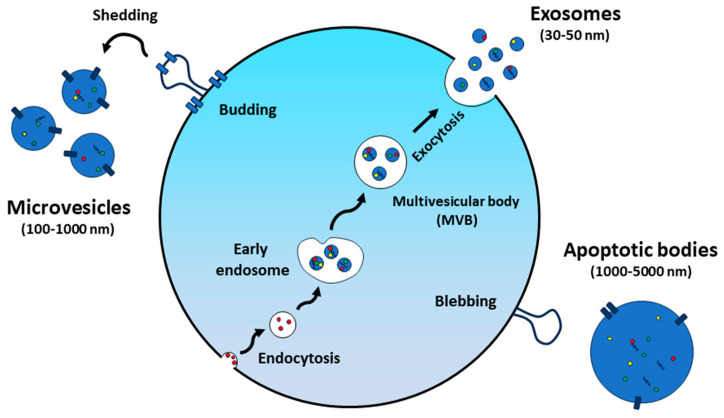
Extracellular vesicle formation and secretion.

**Figure 2:**
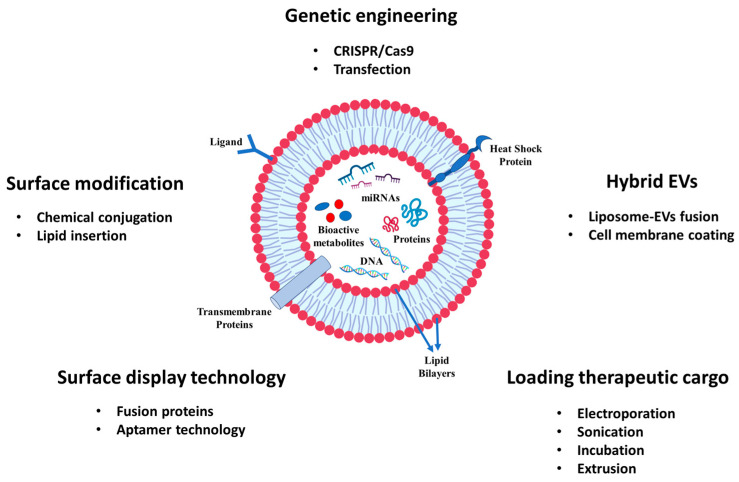
The engineering and modification of EVs for the delivery of cancer drugs and bioactive compounds.

Extracellular vesicles (EVs) are produced by both prokaryotic and eukaryotic cells, typically with sizes ranging from 30 nm to 150 nm (Figure 1). These EVs are spherical-shaped nanoparticles surrounded by a lipid bilayer membrane and were first discovered in 1983 [8]. Although initially identified as cellular waste [9], EVs are now recognized as important mediators of both physiological and pathological intercellular communication, exerting profound effects on a wide range of biological processes [10,11]. They can transport various cargo molecules, including proteins, nucleic acids, and lipids, allowing them to mediate diverse cellular processes (Figure 2) [12]. In the past decades, most studies on EVs have focused on those of mammalian cell origin. In particular, mesenchymal stem cell-derived extracellular vesicles (MSC-EVs) have been employed in various research fields, including drug delivery, regenerative medicine, and immunomodulation studies [13,14,15]. Besides many significant findings, the risk of immune rejection and concerns about potentially carrying harmful substances, such as tumor-derived molecules, infections, or undesirable traits from donor cells to EVs, still remain as significant hurdles for recipients [16]. When it comes to their application in biomedicine, the authorization and scale-up production of mammalian cell-derived EVs will fundamentally depend on factors associated with source and ethical considerations [17,18]. 

Recently, there has been a surge of interest in utilizing EVs derived from plants and fruits for advanced cancer treatments. These unique vesicles, referred to as “plant-derived extracellular vesicles (PDEVs)” or “plant-derived exosome-like particles”, possess a variety of bioactive compounds similar to those of mammalian cell-derived EVs. Plant extracts are known to possess anti-cancer effects and to reduce toxicity associated to standard chemotherapy. For example, many botanical compounds, including curcumin and quercetin, are capable of ameliorating several side effects via modulating the Nrf2, MAPK, and NF-κB signaling pathway, as well as scavenging free radicals [6]. Therefore, they can enhance intracellular antioxidant capacity, inhibit the inflammatory response, and reduce the risk of secondary cancer. A number of phytochemicals, including flavonoids, coumarins, and phenolic compounds, possess antioxidant, anti-inflammatory, immunomodulatory, and even anti-carcinogenic activities. While both PDEVs and plant extracts may offer therapeutic benefits, EVs have unique advantages to encapsulate and deliver bioactive molecules in a targeted and efficient manner. EVs provide a natural and stable packaging for active compounds, protecting them from degradation. Their nanoscale size allows for enhanced cellular uptake, promoting better bioavailability and therapeutic efficacy compared to larger extract particles. PDEVs, with their natural origin, cargo content, and compatibility with biological systems, stand out as an attractive candidate for therapeutic applications [19,20]. The utilization of PDEVs extends beyond addressing inflammation, tissue repair, and gut microbiota modulation, as it is expected to transform cancer therapy by affecting key biological processes such as tumor cell proliferation, apoptosis, and metastasis [21,22]. Despite this treatment approach still being under research, it has already yielded some encouraging research outcomes in the field of oncology across various cancer types [23,24,25]. Notably, the application of PDEVs is not limited to pre-clinical studies and is advancing into clinical trials [26,27], marking a significant step forward in the quest for cancer therapies. 

In this review, we will discuss the diverse array of plant and fruit species that serve as sources for PDEVs, aiming to gain insights into the potential of PDEVs to shape the future of cancer treatment. The latest research on PDEVs in cancer therapy will be explored, including their isolation and characterization methods, cargo-loading capabilities, therapeutic potential, as well as their applications in pre-clinical and clinical oncology settings.

## 2. Isolation and Characterization of PDEVs from Plants and Fruits 

PDEVs can be obtained from a variety of plant sources, including fruits, vegetables, and medicinal plants. The isolation methods typically involve the homogenization of plant tissues, followed by a series of centrifugation and filtration steps to extract and purify PDEVs. A range of diverse methods have been devised to efficiently isolate EVs, including differential ultracentrifugation, precipitation, filtration, immunoaffinity technique, microfluidic separation, and size exclusion chromatography (SEC) [28]. Nonetheless, ultracentrifugation, widely recognized as the “gold standard” for EV isolation [29], remains the predominant method of choice for extracting EVs from plant and fruit sources, due to its consistent ability to yield a relatively high quantity of PDEVs of good quality. Sucrose-based methods are widely used to isolate PDEVs as they produce more purified EVs and prevent protein aggregation contaminants. Prefiltration utilizing a range of filter pore sizes (e.g., 0.2 μm, 0.4 μm, and 0.8 μm) is often used as a preparatory measure before ultracentrifugation procedures. Coupling filtration with sucrose-based ultracentrifugation techniques has arisen as a remarkably efficient strategy for the isolation of EVs from plant and fruit sources. Both sucrose density gradient [30] and sucrose cushion [31,32] ultracentrifugation have demonstrated their proficiency in successfully isolating EVs from various plant origins, including *Panax Ginseng*. Furthermore, these methodologies have been instrumental in the extraction of EVs from a spectrum of plant sources, such as tea flowers [25], *Citrus limon* L. [24], kiwifruit [33], and broccoli [34]. 

These isolated PDEVs need characterization for their intended applications, involving a diverse range of analytical techniques. Physical properties are typically assessed using transmission electron microscopy (TEM), scanning electron microscopy (SEM), cryo-electron microscopy (cryo-EM), and atomic force microscopy (AFM) to determine their dimensions and morphological features. Concentrations and size distributions of PDEVs are commonly determined using dynamic light scattering (DLS) and nanoparticle tracking analysis (NTA) [35]. Furthermore, the confirmation and assessment of their presence and protein composition involve techniques such as bicinchoninic acid (BCA) assay, Western blotting, enzyme-linked immunosorbent assay (ELISA), mass spectrometry, and flow cytometry [36]. 

## 3. Therapeutic Applications of PDEVs in Multiple Types of Cancer 

Over the past few decades, PDEVs have received extensive attention for their role as innovative and promising natural nano-biomedicines with a wide range of potential therapeutic applications. The current literature has proven that such PDEVs can effectively enter mammalian cells and regulate gene regulation through cross-kingdom reactions, thereby interfering with the biological functions of the human body [37]. Their intrinsic therapeutic properties are (1) their antioxidant efficacy for oxidative stress-related diseases [38], (2) the capability to treat autoimmune diseases [39] and regulate immune function and inflammatory responses [40], and (3) their facilitation of tissue regeneration [41], and wound healing [42]. PDEVs also have the ability to modulate gut microbiota, playing a role in mediating gastrointestinal disorders and maintaining gut homeostasis [43]. Additionally, agri-food wastes have been found to contain valuable biomolecules that can act as reducing and capping agents in nanoparticle synthesis. The sustainable green synthesis of silver nanoparticles using EVs derived from agri-food waste and by-products highlights the therapeutic potential of these EVs, particularly their ability to enhance the antibacterial and anti-cancer properties of silver nanoparticles [44]. Ultrasonic-assisted extraction (UAE) was successfully used to effectively extract polyphenols with antioxidant properties from pecan shell waste biomass [45]. Antioxidant capacity, erythrocyte protection, and phenolic content of safflower (*Carthamus tinctorius* L.) by-products were identified by using ultra-high-performance liquid chromatography–diode array detector–tandem mass spectrometry (UPLC-DAD-MS) [46]. These approaches offer an eco-friendly alternative to conventional synthesis, reducing environmental pollution while harnessing the therapeutic potential of PDEVs. 

To date, PDEVs have demonstrated excellent anti-cancer properties in multiple types of cancer, including the inhibition of cancer cell proliferation, induction of apoptosis, and the alteration of the tumor microenvironment in various types of cancer. A detailed overview of the current applications of EVs derived from plants and fruits in cancer therapy is summarized in Table 1. 

### 3.1. Citrus Fruits 

Citrus fruits, including oranges, lemons, limes, grapefruits and tangerines, are known for their high content of vitamin C, antioxidants, and dietary fiber [47]. In 1999, Kawaii et al. showed the anti-proliferative activity of a large group of Citrus fruits, including *C. bergamia*, *C. limon*, *C. grandis*, *C. paradisi*, *C. aurantium*, *C. sinensis*, *C. nobilis*, *C. unshiu*, *C. reticulata*, *C. tangerine*, and *C. clementina* on different cancer cell lines. Despite differences in efficacy, all the tested juices demonstrated antiproliferative activity, with the most pronounced effect observed in *C. nobilis* [48]. Since then, there have been numerous studies conducted to evaluate the use of EVs obtained from citrus fruits for therapeutic purposes. Notably, EVs sourced from lemons have demonstrated remarkable promise in effectively inhibiting tumor progression in a wide range of cancer types. Yang et al. reported lemon-derived extracellular vesicles (LDEVs) induced S-phase cell cycle arrest in three gastric cancer cell lines (AGS, BGC-823, and SGC-7901) and triggered apoptosis through the production of reactive oxygen species (ROS) in vitro. In subsequent in vivo examinations, by using the SGC-7901 xenograft models, LDEVs demonstrated significant efficacy in inhibiting tumor growth, and no histological abnormalities were detected in major organs during safety assessment, confirming its safety [23]. As members of the citrus family, EVs derived from *C. paradisi* and grapefruit showed significant cytotoxic effects on different tumor cell lines. Upon treatment with EVs derived from *C. paradisi* fruit juice at a concentration of 25 μg/mL, a significant decrease in cell viability was observed in A375 human melanoma cells. Regarding grapefruit, A375 and MCF7 breast cancer cells exhibited the highest responsiveness, with more than a 30% reduction in viability. More specifically, grapefruit EVs led to cell cycle arrest and the significant downregulation of the expression of the phosphorylated ERK and AKT. Additionally, these EVs effectively reduced the expression levels of intercellular adhesion molecule 1 (ICAM1) and the cysteine protease cathepsin, two key mediators in the process of cancer progression [49]. In other studies, Raimondo and colleagues demonstrated that the EVs from lemon juice (*Citrus limon* L.) exhibit inhibitory effects on the proliferation of various cancer cell lines, such as A549, SW480, and LAMA84, and suppress tumor growth in a tumor xenograft model by inducing TRAIL-mediated apoptotic cell death [24]. After this study, they conducted proteomic profiling of human colorectal adenocarcinoma cells (SW480) to unravel the mechanism of action underlying EVs’ effects. The investigation revealed that the reduced growth of cancer cells resulted from the inhibition of lipid metabolism, via the downregulation of Acetyl-CoA Carboxylase 1 (ACACA) [50]. 

### 3.2. Other Fruits 

Romina strawberry or its anthocyanin fraction showed great efficacy in inhibiting extracellular matrix (ECM) components, including collagen 1A1, fibronectin, activin A, and proteoglycan versican in both primary myometrial and leiomyoma cells [51]. Grape exosome-like nanoparticles (GELNs) have been shown to induce intestinal stem cell proliferation and have protective effects against dextran sulfate sodium (DSS)-induced colitis in mice [41]. 

The oral administration of GELNs to mice significantly promoted the proliferation of Lgr5-EGFP+Ki67+ intestinal stem cells. By culturing intestinal crypts in 3D, it was confirmed that GELNs enhance the proliferation of Lgr5-EGFP^hi^ stem cells, which is essential for creating long-lasting intestinal organoids and accelerating the development of these organoid structures. Liposome-like nanoparticles assembled with lipids from GELNs proved critical for targeting and inducing intestinal stem cells. Moreover, the increase in β-galactosidase + (β-Gal) intestinal crypts in B6.Cg-Tg (BAT-lacZ) 3Picc/J mice suggests that GELN-based treatment of mice enhances Wnt-mediated activation. Real-time PCR analysis of EGFP^hi^ cells from the crypts of Lgr5-EGFP-IRES-CreERT2 mice revealed the upregulated expression of the genes related to stem cell growth, including AXIN-2, Cyclin D1, c-Myc, and EGFR. Notably, daily administration of GELNs (2 mg per mouse) prevented the progression of DSS-induced colitis. Bitter melon (*Momordica charantia*) extract has demonstrated anti-cancer effects against a variety of tumors [52,53,54,55]. Building on this, Yang et al. isolated bitter melon-derived extracellular vesicles (BMEVs) from bitter melon juice and investigated their synergistic therapeutic efficacy with 5-fuorouracil (5-FU) against oral squamous cell carcinoma (OSCC) [56]. Upon the co-administration of 50 mg/kg BMEV + 5-FU, the tumor size of mice in this group was significantly reduced compared with the mice in the group given 5-FU and BMEV alone. Moreover, given that the development of chemoresistance in OSCC cells to 5-FU was attributed to the aberrant expression and activation of the NOD-like receptor family pyrin domain-containing 3 (NLRP3) inflammasome, the expression levels of NLRP3 were measured for the group of mice treated with the combination of BMEV + 5-FU and 5-FU alone. The results showed the downregulated expression of NLRP3 and IL-1β in tumors derived from the group of mice treated with the combination of BMEVs + 5-FU, as compared to mice administered 5-FU alone. 

### 3.3. Ginseng 

Among plant sources, *Ginseng* EVs have attracted considerable attention as one source of traditional herbal medicine, their therapeutic applications not limited only to cognitive functions, cardiovascular diseases, but extending towards cancer treatment. According to the study conducted by Cao et al., EV-like *Panax ginseng C.A. Mey*.-derived nanoparticles (GDNPs) proficiently inhibit melanoma growth by inhibiting the M2-like polarization of macrophages through a mechanism dependent on Toll-like receptor (TLR)-4/myeloid differentiation antigen 88 (MyD88) signaling. In the experimental setting involving C57/BL6 mice bearing melanoma tumors, those treated with GDNPs exhibited a significant decrease in tumor growth [57]. Another group of studies demonstrated that ginseng-derived exosome-like nanoparticles (GEN) can penetrate the blood–brain barrier (BBB). This unique property enables these nanoparticles to produce significant therapeutic effects [32]. Such nanoparticles suppress bone marrow-derived macrophage (BMM) polarization towards M2 and promote the expression of M1 macrophages within the tumor microenvironment (TME) by modulating cancer-associated fibroblasts (CAFs). In mice administered with GENs via intravenous delivery, no discernible evidence of liver toxicity was observed. These findings have brought to light the therapeutic efficacy of *ginseng*-derived EVs as nanodrugs for advancing cancer treatment. 

### 3.4. Ginger 

Ginger (*Zingiber officinale*), a member of the Zingiberaceae family, is a plant that produces a rhizome called ginger root. This root has been used in various Asian countries for hundreds of years and enjoys a high status in traditional medicine and is widely used for its healing properties [58]. Recent studies have clearly demonstrated that ginger-derived EVs (GDEVs) possess significant anti-inflammatory [59,60] and anti-cancer capabilities [61]. Of particular interest is the study conducted by Zhang et al., which successfully demonstrated the anti-cancer properties of these EVs in colitis-associated cancer (CAC) [62]. DSS-induced colitis mice that received ginger-derived nanoparticles (GDNPs) orally exhibited substantial anti-inflammatory responses. This was marked by a decrease in the levels of pro-inflammatory cytokines, including TNF-α, IL-6, and IL-1β. Concurrently, there was an increase in the production of anti-inflammatory cytokines such as IL-10 and IL-22. These alterations in cytokine levels were effective in preventing colitis induced by dextran sulfate sodium (DSS). In IL-10 knockout (IL10−/−) mice, the GDNP treatment showed anti-inflammatory activity that was able to prevent chronic colitis. In mice with azoxymethane/dextran sulfate sodium (AOM/DSS) colorectal cancer (CRC), daily oral administration of 0.3 mg GDNPs significantly reduced IL-6 and IL-1β levels, as well as the mRNA expression of TNF-α and cyclin D1. These studies reveal that the therapeutic potential of GDEVs appears promising, providing a new avenue for creating natural, bioinspired treatment options. 

### 3.5. Tea Leaves and Flowers 

Tea, abundant in antioxidants—especially polyphenols—acts as a protective shield against free radicals, thereby reducing the risk of chronic diseases [63]. Zu et al. discovered that nanotherapeutics (NTs) derived from tea leaves effectively inhibit the progression of colitis-associated cancer (CAC) [64]. The RAW 264.7 macrophages upon treatment with NTs, showed a downregulation in the production of pro-inflammatory cytokines such as TNF-α, IL-6, and IL-12, along with an upregulation in the secretion of the anti-inflammatory cytokine IL-10. This suggests that tea leaf-derived NTs have preventive capabilities against inflammatory diseases. As described in the following phase of the study, mice were orally administered daily with 2 mg/kg of NTs during the dextran sulfate sodium (DSS) treatment period. The animals effectively prevented hepatosplenomegaly, preserved the morphological integrity of major organs, and significantly reduced the number of tumors per mouse. This suggests that the oral administration of NTs exhibits excellent biocompatibility while effectively mitigating inflammatory responses and inhibiting colon tumor development. Tea flowers (*Camellia sinensis* L.) contain similar functional components to those found in tea leaves [65]. Their extracts have shown effective anti-cancer properties in several types of cancer, including breast cancer [66], ovarian cancer [67], and non-small cell lung cancer [68]. Recently, tea flower-derived extracellular nanoparticles (TFENs) have shown great promise in inhibiting breast tumors as well as lung metastasis [25]. Obvious anti-proliferation activities of TFENs were observed in MCF-7 cells, 4T1 cells, A549 cells, and HeLa cells. The groups of mice that received TFENs by oral and intravenous (i.v.) injection achieved comparable therapeutic effects. Considering that most breast cancer tends to metastasize to the lung, the therapeutic efficacy of TFENs on lung metastasis were investigated by the authors. Studies showed they inhibited the tumor growth in breast cancer and metastasis of lung carcinomas in xenograft tumor models via reactive oxygen species generation and microbiota modulation. 

**Table 1:** Intrinsic therapeutic potential of PDEVs from different plants and fruits.

PDEVs	Type of Study	Type of Cells and/or Mice Models	Results	Ref.
Grape fruits and *C. paradisi*	In vitro	A375 human melanoma cell line, MCF7 breast adenocarcinoma cell line, A549 human lung carcinoma cell line.	Grapefruit-derived EVs, alongside those from *C. paradisi*, exhibited strong cytotoxicity against tumor cells, especially A375 melanoma cells, resulting in substantial viability reduction, cell cycle arrest, the downregulation of phosphorylated ERK and AKT, and a decrease in key mediators (ICAM1 and cathepsin) associated with cancer progression.	[49]
Lemon-derived extracellular vesicles (LDEVs)	In vitro and in vivo	Gastric cancer cell line AGS, BGC-823, and SGC-7901. SGC-7901 xenograft models.	LDEVs induced S-phase cell cycle arrest and apoptosis in gastric cancer cells through the generation of ROS in vitro and suppressed gastric cancer growth in vivo with no toxicities in major organs.	[23]
*Citrus limon juice* L.-derived nanovesicles	In vitro and in vivo	A549 human lung carcinoma cell line, SW480 human colorectal adenocarcinoma cell line, LAMA 84 human chronic myeloid leukemia cell line. LAMA84 xenograft models.	*Citrus limon juice* L.-derived nanovesicles inhibit the proliferation of diverse cancer cell lines in vitro and suppress tumor growth in vivo by inducing TRAIL-mediated apoptotic cell death.	[24]
Romina fruit and its anthocyanin fraction	In vitro	Patient-derived primary myometrial and leiomyoma cells.	Romina (R) and Romina anthocyanin (RA) treatment significantly inhibited the expression of ECM components including collagen 1A1, fibronectin, and versican in leiomyoma cells.	[51]
Grape exosome-like nanoparticles (GELNs)	In vitro and in vivo	Intestinal epithelial CT26 cell line, Crypt cells, EGFP^hi^ cells, and Lgr5-EGFP^+^ cells. Lgr5-EGFP-IRES-CreERT2 mice, B6.Cg-Tg(BAT-lacZ)3Picc/J mice.	The oral administration of GELNs can promote intestinal stem cell proliferation and organoid formation, stimulates Wnt/β-catenin pathway activation, and enhances the expression of stem cell growth-related genes. Lgr5-EGFP-IRES-CreERT2 mice were effectively prevented from DSS-induced colitis at a daily dose of 2 mg.	[41]
*Panax ginseng C.A. Mey*.-derived nanoparticles (GDNPs)	In vitro and in vivo	Murine melanoma cell line (B16F10), breast cancer cell line (4T1), and human embryonic kidney cell line (HEK293T). MyD88-, TLR4-, and TLR2-deficient C57/BL6 mice.	GDNPs altered M2 polarization and significantly suppressed melanoma growth in vitro and in vivo in tumor-bearing mice.	[57]
*Panax ginseng*-derived exosome-like nanoparticles (GENs)	In vitro and in vivo	C6 rat glioma cells, mouse embryonic fibroblast NIH3T3 cells, brain capillary endothelial cells (BCEC). Male Wistar rats (8 weeks old, 200–250 g) and male Balb/C mice (6–8 weeks old, 18–20 g).	GENs penetrated the blood–brain barrier (BBB) in C6 glioma cells and accumulated in mice brain tumors. The intracranial (IC) and intravenous (IV) injection of GENs effectively inhibited tumor growth in mice. GENs with anti-glioma effects can influence the tumor microenvironment (TME) by regulating tumor-associated macrophages (TAMs).	[32]
Ginger-derived nanovesicles (GDNs)	In vitro and in vivo	RAW 264.7 cells, Caco-2BBE, and Colon-26 cells. DSS-induced colitis mouse model, IL-10 knockout (IL10−/−) mice, and chemically induced colorectal cancer (CRC) models.	GDNPs did not affect cell viability, and do not cause local or systemic side effects. Anti-inflammatory effects were observed by increased levels of anti-inflammatory cytokines and decreased levels of pro-inflammatory cytokines.	[62]
Tea leaf-derived nanotherapeutics (NTs)	In vitro and in vivo	RAW 264.7 macrophages. C57BL/6 mice (12 weeks of age).	Treatment with tea leaf-derived NTs reduced pro-inflammatory cytokines and increased anti-inflammatory cytokines in RAW 264.7 macrophages. The oral administration of tea-derived NTs to mice during DSS treatment showed excellent biocompatibility, protected organs, and significantly reduced colon tumors.	[64]
Tea flowers-derived exosome-like NPs (TFENs)	In vitro and in vivo	MCF-7 cells, 4T1 cells, A549 cells, and HeLa cells Human breast cancer MCF-7 xenograft tumor model and Lung metastasis mice model	The anti-proliferative effects of TFENs resulted in mitochondria damages in MCF-7 and 4T1 cells and triggered cell cycle arrest. A significant inhibition of breast tumor growth and mitigation of lung metastasis were observed in mice administered intravenously or orally at doses of 1.5 or 3 mg TFENs/kg.	[25]
Bitter melon-derived extracellular vesicles (BMEVs)	In vitro and in vivo	WSU-HN6 and CAL27 oral squamous cell carcinoma (OSCC) cell line. Female BALB/c nude mice (4–6 weeks old).	The synergistic effect of BMEV + 5-FU resulted in the downregulation of NLRP3 and IL-1β expression in mouse OSCC tumors.	[56]

## 4. Nano-Delivery Systems of PDEVs for Cancer Treatment

PDEVs not only function as therapeutics in their own right but have also gained recognition as ideal vehicles for the delivery of therapeutic agents. Their potential lies in their ability to act as a natural delivery system. This facilitates the targeted delivery of therapeutics to tumor sites while minimizing off-target effects and reducing systemic toxicity. The inherent biocompatibility and biodegradability of PDEVs further enhances their attractiveness as a delivery platform, potentially overcoming the limitations associated with synthetic nanoparticles [69]. These vesicles have been shown to encapsulate a variety of molecules, including small RNAs, proteins, and lipids, some of which play pivotal roles in cancer-related processes, induce apoptosis in cancer cells, inhibit cancer cell growth, and exert control over the tumor microenvironment. Certain plant-derived microRNAs (miRNAs) and small interfering RNAs (siRNAs) have been shown to silence oncogenes or activate tumor suppressor genes, while specific proteins and peptides can induce apoptosis in cancer cells. This section will discuss in detail the anti-cancer properties of PDEVs when carrying specific cargo molecules, including therapeutics, miRNAs, siRNAs, and anti-inflammatory agents (Table 2). 

### 4.1. Grapefruit 

Grapefruit-derived EVs have attracted considerable interest as vehicles for the targeted delivery of therapeutics to specific tumor sites. Wang and his colleagues applied grapefruit-derived nanovectors (GNVs) for drug delivery, demonstrating their promising potential [70]. These GNVs exhibit a high capacity to be taken up by various cell types, including primary lymphocytes, with minimal toxicity, and have been shown to efficiently transport biotinylated substances, including DNA and proteins, to target cells. Biotinylated enhanced Yellow Fluorescent Protein (eYFP) DNA vectors carried by GNVs achieved YFP expression in A549 cells as effectively as traditional transfection methods like Lipofectamine 2000, highlighting the GNVs as excellent delivery vectors for therapeutics. In the in vivo settings, groups of GL26 tumor-bearing mice administered intranasally with GNVs encapsulating the Stat3 inhibitor JSI-124 showed significant inhibition of tumor growth compared to the other groups of mice that received phosphate-buffered saline (PBS) or GNVs alone. Furthermore, tumor-biasing properties were confirmed by the two tumor xenograft models, including the mouse CT26 colon cancer model and the human SW620 colon cancer SCID mouse model. Mice that received GNVs-folic acid (FA)-paclitaxel (PTX) intravenously showed enhanced targeted delivery and a substantial decrease in tumor growth compared to those that received GNVs alone in both independent murine cancer models. Importantly, GNVs did not cause any adverse effects or pathological changes in major organs such as the lung, kidney, liver, or spleen, demonstrating their safety in treated animals when compared to those that did not receive any treatment. Following this study, the authors went on to investigate the use of GNVs coated with activated leukocyte membranes for the targeted delivery of therapeutic agents to inflammatory tumor sites [71]. These GNVs, named inflammatory GNVs (IGNVs), show enhanced homing abilities to inflammatory tissues in various inflammatory mouse models and tumor-bearing mouse models. The administration of doxorubicin-loaded IGNV-DOX resulted in a higher concentration of DOX in tumors and lower levels in the liver compared to mice treated with DOX-NP^™^. This targeted delivery was confirmed by more intense DOX signals in both CT26 colon and 4T1 breast tumors, leading to significant tumor growth inhibition and prolonged survival of the mice. Moreover, IGNVs loaded with curcumin (IGNV-Cur) were more effective in reducing colitis than other treatments. These studies highlighted the enhanced therapeutic effects of IGNVs in cancer and inflammatory diseases. Echoing the results of this study, research from other groups has shown that anti-inflammatory agents carried by grapefruit-derived nanovesicles (GDNs) can improve immunomodulatory effects and enhance host immune responses [72]. They used GDNs for targeted drug delivery to intestinal macrophages, aiming to treat inflammatory bowel diseases such as Crohn’s disease and ulcerative colitis. GDNs selectively targeted intestinal macrophages, and enhanced their anti-inflammatory response by upregulating heme oxygenase-1 (HO-1) and IL-10, and inhibiting the secretion of inflammatory cytokines, TNF-α and IL-1β, thereby ameliorating colitis in mice. The oral delivery of GDNs encapsulating methotrexate (MTX), an immunosuppressive and anti-inflammatory agent, selectively targets lamina propria macrophages and significantly enhances the anti-inflammatory effects of MTX in a DSS-induced mouse colitis model. The study highlights the potential of GDNs as attractive strategies for the oral delivery of anti-inflammatory drugs to prevent or treat autoimmune diseases and colon cancer. 

### 4.2. Other Fruits 

The lipid composition of PDEVs can be tailored to enhance membrane fusion with target cells, thereby improving the efficacy of cargo delivery. Recently, orange-derived extracellular vesicle (OEV) nanodrugs (DN@OEV), which modified by attaching cRGD-targeted doxorubicin (DOX) nanoparticles (DN) onto the surface of OEV, have shown promising anti-cancer effects against ovarian cancer [73]. This strategic modification improved transcytosis effects, and achieved targeted delivery to ovarian cancer cells. The intraperitoneal injection of DN@OEV in orthotopic SKOV3-Luc bearing ovarian cancer nude mice resulted in enhanced tumor accumulation and penetration, significantly inhibiting tumor growth and metastasis compared to free DOX and DN. Fang et al. recently discovered that kiwifruit-derived extracellular vesicles (KEVs) offer significant potential as oral delivery vehicles for the lipophilic multi-targeted kinase inhibitor sorafenib (SFB) in cancer treatment [33]. These KEVs-SFB are stable in the gastrointestinal tract and mainly accumulated in the liver of the orthotopic HepG2 liver cancer xenograft nude mice. Their anti-tumor properties were demonstrated both in vitro and in vivo, leading to the significant inhibition of cell proliferation and a notable reduction in tumor growth in mice. Furthermore, the nontoxic properties of KEVs toward normal hepatocytes suggest they may be an effective option for treating liver cancer with reduced side effects. 

### 4.3. Ginger 

Numerous studies have identified ginger-derived EVs as effective vectors for transporting therapeutic agents, including miRNA, siRNA, and DOX. The study conducted by Li. et al. utilized ginger-derived exosome-like nanovesicles (GDENs) for the systemic delivery of siRNA for cancer suppression [74]. GDENs were engineered to present FA as a targeting ligand on their surface, facilitating the precise delivery of survivin siRNA to tumors. FA-conjugated arrowtail pRNA-3WJ enhanced the binding of GDENs to KB cells and facilitated the uptake of GDENs by KB cells. The resulting complex of FA-3WJ/GDENs/siRNA demonstrated markedly lower toxicity compared to traditional transfection agents like Lipofectamine 2000, highlighting its superior biocompatibility as a delivery system. When administered intravenously in KB cell xenograft mice, the GDENs effectively inhibited tumor growth. Another research group also succeeded in delivering siRNAs using ginger-derived lipid vehicles (GDLVs) [75]. siRNA-CD98-loaded GDLVs efficiently decreased CD98 expression in colon-26 and RAW 264.7 cells. For six-week-old female FVB mice, the oral delivery of siRNA-CD98/GDLVs showed precise targeting to the large intestine, significantly diminishing CD98 expression in the ileum and colon—key areas affected by ulcerative colitis—without affecting other parts of the digestive tract. These outcomes indicated the in vivo effectiveness of GDLVs in delivering siRNA, highlighting its biosafety, precision, and potential therapeutic value. Prior to this study, this group of researchers successfully loaded DOX into ginger-derived nanocarriers (GDNVs) and determined their stability and ability to release the drug in a pH-dependent manner. This highlights their potential for targeted drug delivery to the acidic tumor microenvironment [62]. GDNVs were shown to be efficiently taken up by Colon-26 and HT-29 cancer cells through endocytosis, particularly via the phagocytosis pathway, and were found to be non-toxic in vivo. Additionally, DOX-loaded GDNVs (DOX-GDNVs) exhibited pronounced cytotoxic activity against Colon-26 and HT-29 cells in vitro, and FA-GDNVs-loaded DOX (DOX-FA-GDNVs) precisely targeted tumors and dramatically inhibited tumor growth and reduced tumor volume in a Colon-26 xenograft mouse model. The treatment elicited no obvious histopathological damage in major organs, indicating that GDNVs may mitigate the systemic toxicity and adverse reactions commonly linked to free DOX administration. 

### 4.4. Other Plants

Broccoli-derived EVs have demonstrated their potential as innovative nanocarriers for exogenous miRNA delivery [76]. These EVs can protect miRNAs from RNase degradation and gastrointestinal digestion, and exhibited 30% cytotoxicity in Caco-2 cells. As a natural source, their low immunogenicity and stability in the gastrointestinal tract make them a promising candidate for RNA-based therapeutic applications. Zeng et al. conducted research on the use of aloe-derived nanovesicles (ADNVs) as nanocarriers for encapsulating and delivering indocyanine green (ICG) in phototherapy, specifically targeting cancer treatment. Their research compared two types of ADNVs extracted from aloe gel (gADNVs) and rind (rADNVs). The investigation into their stability and antioxidant capacities revealed that gADNVs, which are rich in antioxidant components, displayed enhanced stability and preserved their structural integrity even without the addition of stabilizers. The safety profiles of ADNVs were assessed through in vitro and in vivo studies, revealing no significant toxicity in B16F10, MCF-10A, and 4T1 cell lines, nor in mice administered with gADNVs and rADNVs intravenously. Observations indicated no hemolysis and a lack of toxicity to erythrocytes in mice treated with gADNVs. Moreover, no noticeable organ damage was detected in either the gADNVs or Lips treatment groups. Indocyanine green (ICG)-loaded gADNVs (ICG/gADNVs) exhibited superior stability and leak-proof capabilities than Lips, establishing them as more effective nanocarriers for drug delivery. These ICG/gADNVs demonstrate dose-dependent cytotoxic effects in vitro and remarkable efficacy in tumor suppression in vivo, with their therapeutic effectiveness sustained for an extended period, notably beyond 30 days. These findings emphasize the potential of gel-derived Aloe nanovesicles (gADNVs) as a cost-effective and non-toxic alternative for drug delivery systems, with potential applications in noninvasive transdermal administration and skin cancer therapy. 

**Table 2:** Delivery system of PDEVs in cancer treatment.

ORIGINS	Type of Study	Type of Cells and/or Mice Models	Findings	Ref.
Grapefruit-derived nanovectors (GNVs)	In vitro and in vivo	Mouse glioma 261 (Gl261) cells, murine mammary carcinoma cell line 4T1, lung carcinoma cell line A549, murine colorectal carcinoma cell line CT26, human colorectal adenocarcinoma cells SW620, and primary lymphocytes. C57BL/6j mice, BALB/c mice, and NOD/SCID mice 6–8 weeks of age.	GNVs, as therapeutic delivery vectors, transport biotinylated molecules and achieve targeted gene expression with minimal toxicity. The intranasal injection of GNVs encapsulated with the Stat3 inhibitor JSI-124 into GL26 tumor-bearing mice showed a significant inhibition of tumor growth. The intravenous administration of GNVs-folate (FA)-paclitaxel (PTX) showed enhanced targeted delivery and a significant reduction in tumor growth in the mouse CT26 colon cancer model and the human SW620 colon cancer SCID mouse model. No adverse reactions or major organ lesions were observed.	[70]
Grapefruit-derived nanovectors (GNVs)	In vitro and in vivo	Mouse T lymphoma EL4 cells, mouse 4T1, 4TO7 breast cancer cell lines, mouse NMuMG mammary gland epithelial cells, CT26 colon cancer and human umbilical vein endothelial cells (HUVECs), and CT26 cells. DSS-induced colitis mice; CT26 tumor model and 4T1 tumor model.	IGNVs exhibit advanced homing properties to inflammatory tissues. IGNV-DOX was highly accumulated in the tumor site and the I.V. administration of IGNV-DOX to the mice led to notable decreases in the growth of breast and colon tumors in tumor-bearing mice. In mice with DSS-induced colitis, IGNV-Cur treatment showed better therapeutic efficacy in inhibiting colitis than curcumin-loaded GNV or curcumin alone.	[71]
Grapefruit-derived nanovesicles (GDNs)	In vitro and in vivo	Macrophages, intestinal epithelial cells, intestinal leukocytes, RAW264.7 cells. DSS-induced colitis mouse model.	Orally administered GDNs deliver methotrexate (GMTX) to intestinal macrophages, significantly reducing MTX toxicity while enhancing its anti-inflammatory effects in a DSS-induced mouse colitis model.	[72]
Orange-derived extracellular vesicle-based nanodrugs (DN@OEV)	In vitro and in vivo	Human ovarian cancer cell line SKOV3 and mouse breast cancer cell line 4T-1. Orthotopic SKOV3-Luc ovarian cancer xenograft nude mouse model.	DN@OEV induced an excellent transcytosis process in SKOV3 and 4T-1 cells. Upon intraperitoneal injections, DN@OEV showed high penetration and accumulation in the tumor tissue of mice with orthotopic ovarian cancer, effectively reducing tumor growth and preventing metastasis.	[73]
Kiwifruit-derived extracellular vesicles’ (KEVs) targeted delivery of sorafenib (KEVs-SFB)	In vitro and in vivo	Human hepatocyte LO2 cells, HepG2 human hepatoblastoma cells. Orthotopic HepG2 liver cancer xenograft nude mouse models.	KEVs-SFB remained stable in the gastrointestinal tract and accumulated in the liver after oral administration to orthotopic HepG2 liver cancer xenograft mice. The anti-tumor ability of KEVs-SFB was demonstrated both in vitro and in vivo.	[33]
Systemic delivery of siRNA by ginger-derived exosome-like nanovesicles (GDENs)	In vitro and in vivo	HEK-293, Raw 264.7 macrophages, and KB cells (human epithelial carcinoma cells). KB cell xenograft mice model.	Ligand displayed on the surface of GDENs facilitated cellular uptake, and improved the systemic delivery of siRNAs to the cancer cells. KB cell xenograft mice that received FA-3WJ/GDENs/siRNA complexes via intravenous injection effectively inhibited tumor growth.	[74]
Ginger-derived lipid vehicles (GDLVs)	In vitro and in vivo	Caco-2BBE, Colon-26 cells, and RAW 267.4 macrophages. Six-week-old female FVB mice.	GDLVs loaded with siRNA-CD98 efficiently decreased CD98 expression in colon-26 and RAW 264.7 cells, as well as in the mouse colon, following two oral doses of the siRNA-CD98/GDLV complexes.	[75]
Ginger-derived nanovectors (GDNVs)	In vitro and in vivo	Human Colorectal Adenocarcinoma Stable Cell Line HT29, Caco2-BBE cells, and mouse colon adenocarcinoma cell line colon-26. Colon-26 subcutaneous xenograft model.	GDNVs are taken up by Colon-26 and HT-29 cancer cells via endocytosis. DOX-GDNVs exhibit significant cytotoxic activity against Colon-26 and HT-29 cells in vitro. In the Colon-26 xenograft mouse model, the intravenous injection of DOX-FA-GDNVs effectively targeted tumors and significantly inhibited tumor growth and reduced tumor volume. No obvious histopathological damage to major organs was observed.	[62]
Broccoli EVs loaded with exogenous miRNAs	In vitro	Human colorectal adenocarcinoma cells (Caco-2).	Exogenous miRNA-loaded broccoli EVs protect miRNAs from RNase degradation and gastrointestinal digestion, resulting in 30% cytotoxicity in Caco-2 cells.	[76]
Aloe-derived nanovesicles (ADNVs)	In vitro and in vivo	Murine melanoma cell line B16F10 cells, 4T1 cells, and MCF-10A cells. Male BALB/c mice.	gADNVs possess good stability and antioxidant properties. Their safety profiles were validated both in vitro and in vivo. Moreover, ICG-loaded gADNVs are stable and exhibit dose-dependent cytotoxicity in vitro and significant tumor-suppressive efficacy in vivo.	[77]

## 5. Clinical Trials of PDEVs as Anti-Tumor Agents 

Despite growing enthusiasm, only two clinical trials have been launched to explore the anti-tumor properties of PDEVs (Table 3). The first clinical attempt was started in August 2012, and was focused on evaluating the effectiveness of grape-derived EVs in preventing oral mucositis in patients undergoing chemoradiation treatment for head and neck cancer (ClinicalTrials.gov identifier (NCT number): NCT01668849) [27]. This randomized, parallel-assigned intervention study first measured pain levels associated with oral mucositis, which were assessed weekly during treatment and six months after completion of treatment. Secondary outcomes aim to evaluate the levels of immune biomarkers in blood and mucosal tissue by comparing baseline levels with those obtained at the completion of radiation therapy. In the other clinical trial, the efficacy of plant EVs in enhancing curcumin delivery to normal colon tissue and colon tumors was evaluated (ClinicalTrials.gov identifier (NCT number): NCT01294072) [26]. Curcumin, the active compound found in turmeric, has shown the potential to interfere with colon carcinogenesis and inhibit the growth of colon cancer cell lines in various studies [78]. In this study, participants were divided into three distinct groups. The first group received curcumin alone, the second group received curcumin conjugated with plant EVs, and the third group did not receive any treatment, serving as a control group. Participants were evaluated over a 7-day period following enrollment, with outcomes focused on the concentration of curcumin in normal and cancerous tissue, the safety and tolerability of the treatments, and their effects on colon cells, including immune system response and metabolic characteristics. 

## 6. Advantages and Challenges of PDEVs Compared to MDEVs

EVs have garnered significant attention in cancer research due to their role in mediating communication between tumor cells and their microenvironment, contributing to various aspects of cancer progression, including tumorigenesis, metastasis, angiogenesis, and immune evasion [79]. Among various types of EVs, mammalian cell-derived EVs (MDEVs) have been extensively studied for their potential in therapeutic applications [80]. EVs sourced from mesenchymal stem cells (MSCs), particularly those isolated from human bone marrow (BM) and umbilical cords (UC), were found to inhibit proliferation and promote apoptosis in different types of cancer cells [81]. Furthermore, macrophage-derived EVs have been studied for their potential to reprogram the immune landscape within the TME, potentially enhancing the effectiveness of cancer treatments [82]. Beyond their own capabilities, the versatility of EVs extends to their use as vehicles for the targeted delivery of anti-cancer agents. Originating from a variety of cellular origins, including macrophages, dendritic cells, and MSCs, MDEVs have demonstrated their efficiency in directly transporting anti-cancer therapeutics to tumors. MSC-secreted EVs have been shown to proficiently encapsulate tumor suppressor miRNAs and subsequently undergo targeted release at the tumor site, thereby effectively inhibiting cancer progression [83,84]. Besides the significant outcomes achieved by MDEVs, there are challenges arising from the limited production and the relatively high costs associated with their manufacturing processes. These factors are critical for clinical applications that require standardized dosing and reproducibility. Moreover, MDEVs have been demonstrated to be less immunogenic than cell-based therapies [85]; however, the possibility of eliciting immune responses or transferring undesirable biological substances remains, especially when employing EVs from allogeneic origins or exogenous manipulations. From this perspective, the use of PDEVs for therapeutic research has attracted significant interest over the past decade. Unlike MDEVs, plants can be cultivated at a large scale, yielding a plentiful reserve of EVs for therapeutic purposes. PDEVs, widely regarded as safe and non-toxic, present a minimal risk of containing infectious agents or triggering adverse immune responses. As highlighted in the previous chapters, EVs sourced from plants, such as those derived from lemon [23] or tea leaves [64], are evidenced by their non-toxicity towards major vital organs while selectively targeting tumors, which suggests the excellent safety profile of these PDEVs. In a Phase I clinical trial, grape-derived EVs showed promise in reducing oral mucositis in patients undergoing chemoradiotherapy for head and neck cancer. However, major challenges include ensuring consistency and scalability in the production of EVs. Large-scale plant cultivation and standardized EV isolation are essential. Moreover, the biological behavior of these EVs in vivo is not fully understood, necessitating further research to elucidate their mechanisms of action and long-term effects.

Like mammalian EVs, PDEVs can circulate in the human body, encapsulating and protecting bioactive compounds, such as small RNAs and phytochemicals, which can interact with mammalian cells to affect tumor growth or modulate the tumor microenvironment. Engineered grape-derived EVs have demonstrated effective delivery of chemotherapeutic agents to the targeted site while minimizing off-target effects [70,72]. Additionally, EVs derived from ginger, loaded with siRNAs and chemotherapy drugs, have not only been shown to successfully deliver their cargo to target cancer cells without damaging major organs, but also seem to mitigate the side effects typically caused by chemotherapeutic agents [62,74,75]. The increasing interest in and apparent benefits of PDEVs illuminate their potential as an emerging avenue for tumor therapy. However, the field is still in its early stages, especially when compared to the research on MDEVs. The understanding of their biological behavior in vivo is still limited; more research is required to elucidate their properties and mechanisms of action. Additionally, ensuring their stability and bioavailability and overcoming potential immunogenicity issues are critical for their therapeutic application. With ongoing advancements in research, the expansion of therapeutic applications is anticipated. Overcoming existing challenges will pave the way for PDEVs to emerge as a cornerstone of innovative treatment approaches, offering promising new avenues for drug delivery and treatment strategies, and leading to improved outcomes for patients across a wide array of diseases. 

## 7. Future Perspective

Plant-derived extracellular vesicles (PDEVs) represent an innovative frontier in oncological research, merging the innate advantages of natural derivation, biocompatibility, and precision drug delivery. As our understanding deepens, the potential of PDEVs to revolutionize cancer treatment is becoming increasingly apparent. By taking advantage of the unique properties of PDEVs, the side effects of traditional therapies can be significantly reduced and the effectiveness of treatment improved. Although the road to clinical application is still fraught with a series of challenges, overcoming them is crucial for PDEVs to fully realize their potential as a cornerstone of next-generation cancer treatment.

## 8. Conclusions

Plant-derived extracellular vesicles (PDEVs) have emerged as a promising avenue in the realm of oncological research, owing to the combination of their natural derivation, outstanding biocompatibility, innate tumor-targeting faculties, and adeptness in precision delivery of therapeutic agents. Our intensive review elucidates the burgeoning corpus of evidence underscoring their potential to pivotally advance cancer treatment paradigms. While the current applications of PDEVs in cancer therapy are still being thoroughly investigated, the aggregation of emerging research propels the optimism that they may diminish the adverse effects of conventional cancer treatments. The synthesis of rigorous clinical investigations indicates that PDEVs stand poised to offer a paradigm shift in treatment efficacy, safety, and patient experience [2,3]. Notably, PDEVs enshrine therapeutic agents with profound anti-tumor properties, a characteristic expounded upon in trials that have highlighted their capability to maneuver the cellular microenvironment towards oncogenesis suppression [26,27]. Yet, challenges remain; the yield of PDEVs, their in vivo stability, bioavailability, and potential immunogenicity are pivotal concerns that mandate resolution [23,24,25]. Furthermore, the viability of large-scale production, maintaining batch consistency, and ensuring regulatory compliance are nontrivial hurdles that must be surmounted [19,20]. Addressing these quandaries requires an iterative interplay of experimental research and clinical validation. The empirical data must keep pace with rapidly evolving biotechnological methods, engendering robust processes for the isolation, characterization, and deployment of PDEVs [21,22]. The odyssey from bench to bedside necessitates a tapestry of multidisciplinary collaboration, striving for therapeutic milestones that hinge on the translational potential of PDEVs. 

## Figures and Tables

**Table 3:** Clinical trials of PDEVs for cancer treatment.

PDEVs	Clinical Phase	Intervention/Treatment	Outcome Measures	References
Grape-derived EVs	Phase I	Head and neck cancer	Effectiveness of grape-derived EVs in reducing oral mucositis in patients undergoing chemoradiotherapy for head and neck cancer.	[27]
Plant-derived EVs delivering curcumin	Phase I	Colon cancer tissue	Ability of plant-derived extracellular vesicles to deliver curcumin to normal and colon cancer tissue.	[26]
